# Preparation, Quality Analysis and Antioxidant Activity of Sea Buckthorn (*Hippophae rhamnoides* L.) Kombucha Beverage at Different Fermentation Temperatures

**DOI:** 10.3390/foods14081325

**Published:** 2025-04-11

**Authors:** Yichao Pei, Yuanju Zheng, Michael Yuen, Tina Yuen, Hywel Yuen, Qiang Peng

**Affiliations:** 1 College of Food Science and Engineering, Northwest A&F University, Xianyang 712100, China; peiyichao@nwafu.edu.cn (Y.P.); zyj2022056214@nwafu.edu.cn (Y.Z.); 2 Puredia Limited, Xining 810003, China; michael@puredia.com (M.Y.); tina@puredia.com (T.Y.); hywel@puredia.com (H.Y.)

**Keywords:** kombucha, sea buckthorn, antioxidant activity, sensory evaluation

## Abstract

Sea buckthorn is a unique resource with high nutritional value. The objective of this study was to develop a novel kombucha beverage from sea buckthorn juice by means of inoculation with kombucha (Symbiotic Culture of Bacteria and Yeast, SCOBY). The study investigated and compared the differences in physicochemical properties, antioxidant activity, and sensory evaluation during fermentation at different temperatures with those of traditional cultured green tea kombucha. The findings demonstrated that there were significant variations in physicochemical properties, antioxidant activity, and sensory evaluation among the sea buckthorn kombuchas produced at different temperatures. Among these, the sea buckthorn kombucha produced by fermentation at 28 °C exhibited the strongest antioxidant properties and the most favorable sensory evaluation. Furthermore, changes in the active substances were observed at different temperatures, and correlation analysis revealed that the antioxidant activity of Kombucha tea was correlated with the content of total phenols and total flavonoids. Consequently, the utilization of sea buckthorn juice in the production of kombucha beverages holds considerable promise.

## 1. Introduction

In this day and age, with the ongoing quest for a healthy lifestyle and the growing interest in traditional fermented and natural foods, there is a need for the continued development of new healthy and functional foods to meet this demand [1]. Kombucha, a functional beverage, is produced through the fermentation of sweetened green or black tea. The fermentation process utilizes a SCOBY and produces a plethora of beneficial substances, including probiotics, polyphenols, vitamins, minerals, and organic acids. A plethora of studies have demonstrated the efficacy of kombucha in combating cancer and bacteria, reducing blood pressure, and alleviating cardiovascular disease [2,3]. For instance, Aloulou et al. [4] demonstrated that kombucha possessed the ability to impede the activity of both α-amylase and pancreatic amylase in the blood, thereby attenuating the rise in blood glucose levels. In a similar manner, Cardoso et al. [5] ascertained that kombucha manifested antimicrobial properties, acting upon a multitude of bacteria, including Salmonella, by employing green tea kombucha.

The temperature at which fermentation occurs is a significant indicator of the quality of fermented foods. Maintaining an optimal temperature throughout the fermentation process has been shown to promote microbial growth and enzyme activity, thereby improving the fermentation process. Furthermore, temperature fluctuations have been demonstrated to influence the antioxidant activity of plant-based foods, as evidenced by the production of phenolic compounds [6]. Typically, the temperature range for kombucha fermentation is from 22 °C to 30 °C. However, an alternative approach was adopted by Vitas et al. [7] who utilized an optimization model for the fermentation of dairy products with tea fungi at the following temperature values: 37 °C, 40 °C, and 43 °C. According to their findings, temperature emerged as the predominant factor influencing fermentation duration, with the highest levels of antioxidant activity being attained within the temperature range of 37 °C to 42 °C. In a separate study, Lončar et al. [8] found that higher temperatures resulted in increased production of acids, metabolites, and vitamin C in the samples.

Sea buckthorn (*Hippophae rhamnoides* L.) is a medicinal plant, and sea buckthorn fruit is particularly rich in nutrients and bioactive compounds. Sea buckthorn fruits have been identified as a source of flavonoids, phenolic acids, proanthocyanidins, carotenoids, fatty acids, triterpenoids, vitamins, and phytosterols, as well as other unique bioactive components [9]. A substantial body of research in the medical research domain has demonstrated the efficacy of sea buckthorn berries in reducing cholesterol levels, alleviating angina pectoris, and preventing and controlling coronary atherosclerotic heart disease [10]. Despite the nutritional richness of sea buckthorn, its storage period is brief, one potential solution to mitigate the wastage of resources is to prepare it into fermented food [11].

In conclusion, the present study employed sea buckthorn juice as a fundamental raw material and utilized kombucha fermentation to prepare sea buckthorn kombucha. The study observed the differences in nutrients, physicochemical properties, and other factors under various fermented temperatures. The optimal temperature conditions were identified, providing a valuable reference point for the future commercial development of sea buckthorn kombucha.

## 2. Materials and Methods

### 2.1. Materials and Reagents

The green tea in the kombucha medium was purchased from Trust-Mart Department Store Business (Yangling, China), kombucha (SCOBY) and sea buckthorn juice (diluted 1:1 with deionized water for use) were provided by Puredia Limited (Xining, China), sodium carbonate was purchased from Angel Yeast Co., Ltd. (Yichang, China), sucrose and sodium hydroxide were purchased from Guangdong Guanghua Science and Technology Co., Ltd. (Guangzhou, China), ascorbic acid (Vc) was purchased from Heilongjiang NHU Co., Ltd. (Suihua, China), ABTS and DPPH were obtained from Beijing Boao Toda Technology Co., Ltd. (Beijing, China), and all other drugs used were analytically pure.

### 2.2. Kombucha Production

The preparation was carried out according to the method of Aung and Eun [12] with slight modifications. Firstly, the sucrose medium was prepared by boiling 4 g of green tea in 1 L of distilled water, followed by an extraction period of 5 min, filtration of the residue, and the addition of 80 g of sucrose for dissolution. Following a cooling period to ambient temperature, 25 g of kombucha (SCOBY) was added to each 1 L of medium. The medium was then fermented at 28 °C in conditions of low luminosity for a period of 14 days, until inoculation.

### 2.3. Sea Buckthorn Kombucha (SBK) Fermentation

**Figure 1:**
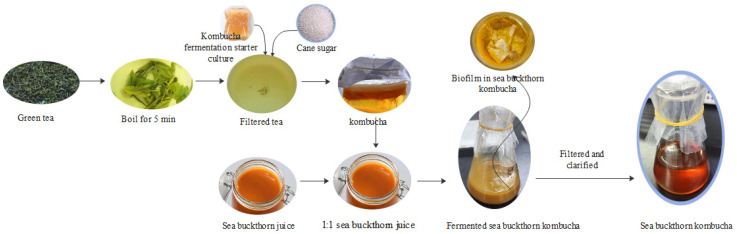
Flow chart of SBK fermentation process.

The pH of the sea buckthorn juice was initially adjusted to 5.0 by the addition of sodium bicarbonate, following which the juice was combined with kombucha tea in a 1:1 ratio. The mixture was then subjected to fermentation by placing it in a container that prevented exposure to light and by operating a shaking apparatus at a rate of 60 rpm. The fermentation temperatures were 20 °C, 28 °C, and 37 °C, which were recorded as K20, K28, and K37 respectively, and the kombucha without sea buckthorn juice was recorded as GKT. The key specific preparation techniques for SBK are illustrated in Figure 1.

### 2.4. Kombucha Quality Analysis

#### 2.4.1. pH and Titratable Acid Determination

The pH was measured in situ using an electronic pH meter (Metrohm Model 827). which had been calibrated at room temperature at pH 4 and 6.18. Titratable acidity was determined by neutralization titration, and determination of total acid content was determined according to national standard GB 12456-2021 [13], which involved the addition of 10 mL of 10-fold diluted samples and the neutralization with 0.05 mol/L NaOH. Phenolphthalein was used as the indicator; the solution turned pink and did not fade within 30 s, indicating was the end point of titration. All tests were repeated three times.

#### 2.4.2. Total Soluble Solids

The total soluble solids (TSS) were measured for all samples using a refractometer (WZS 80, Shanghai, China) and the results were expressed as °Brix values.

#### 2.4.3. Color Analysis

The measurement of kombucha color was performed by utilizing a spectrophotometer according to the method outlined by Zou et al. [14]. This procedure entailed the measurement of the sample’s optical density at increments of 5 nm within the wavelength spectrum ranging from 400 to 700 nm. Distilled water served as a reference standard throughout the experimental process.

The CIELab1931 color parameters were selected and scanned over a range of D65 standard white light source and 2° observer’s field of view conditions. The CIELab color parameters L* (luminance), a* (red-greenness), b* (yellow-blueness), and ∆E*ab (color difference values) were calculated. The color difference between the pre-inoculation tea broth and the fermented sample can be estimated, based on the value of ∆E*. For example, a value below 0.5 is not significant, 0.5~1.5 is slightly significant, 1.5–3.0 is significant, 3.0–6.0 is very significant, and >6.0 is obvious [15].

### 2.5. Determination of Total Polyphenols

The total phenol (TP) content of the samples was determined according to the method of Yang et al. [16]. The reaction was carried out by adding 1 mL of sample to 0.5 mL of Folin–Ciocalteu reagent for 1 min, then adding 1.5 mL of 20% Na_2_CO_3_ and distilled water up to 10 mL. The resultant mixture was then reacted for 10 min in a water bath at 70 °C. Absorbance was measured by UV spectrophotometer at 765 nm.

To prepare the gallic acid standard curve, the specific steps taken were as follows: precision weighing of gallic acid standard, dissolved and fixed to 100 mL, formulated into a 50 mg/L reserve solution; take 0, 0.2, 0.4, 0.6, 0.8, or 1.0 mL of the reserve solution, respectively, into a 25 mL volumetric flask, and add water to 6 mL; add 0.5 mL of Folarin’s reagent to each tube, mix, then add 1.5 mL of 20% Na_2_CO_2_ solution, followed by 1.5 mL of 20% Na_2_CO_2_ solution up to 25 mL; after 10 min of water bath at 75 °C, the absorbance of each gradient was measured at 760 nm with the blank solution (0 mg/L) as reference, and finally the linear equation was fitted with the concentration (0.0, 1.0, 2.0, 3.0, 4.0, 5.0 mg/L) as horizontal coordinates and the absorbance as vertical coordinates (R^2^ ≥ 0.99).

### 2.6. Determination of Total Flavonoids

The total flavonoid (TF) content of the samples was determined according to the method of Dewanto et al. [17]. To determine the total flavonoid (TF) content of samples, 1 mL of sample was added to 0.15 mL 5% NaNO_2_ reagent and allowed to react for 5 min, then 0.15 mL 10% Al(NO_3_)_3_ was further added and allowed to react for 6 min. Then, 2 mL 1 M NaOH was added, up to 10 mL with ethanol, allowed to react for 30 min at room temperature, and the absorbance was measured by UV spectrophotometer at 510 nm.

The specific steps for the preparation of the rutin standard curve were as follows: rutin standard was accurately weighed, dissolved to 100 mL and made into a 1 mg/mL reserve solution; 0, 1.0, 2.0, 3.0, 4.0, or 5.0 mL of the reserve solution were taken into 25 mL volumetric flasks and the final concentration was 0.0–0.2 mg/L; 1 mL 5% NaNO_2_ was added to each tube in turn, allowed to stand for 5 min, then 1 mL 10% Al(NO_3_)_3_ was added, allowed to stand for 6 min, and finally 4 mL 1 M sodium hydroxide was added and allowed to stand with ethanol for 6 min. Then, 1 mL 5% NaNO_2_ was added, allowed to stand for 5 min, 1 mL 10% Al(NO_3_)_3_ was added, allowed to stand for 6 min, and finally 4 mL 1 M sodium hydroxide was added, fixed with ethanol, and allowed to react for 15 min in the dark.

### 2.7. Determination of Antioxidant Activity

#### 2.7.1. DPPH Free Radical Scavenging

The DPPH free radical scavenging activity was analyzed according to the method proposed by Eberhardt et al. [18]. A volume of 1 mL of sample was mixed with 1 mL of 0.1 mmol/L DPPH ethanol solution. The mixture was shaken vigorously and allowed to stand in the dark for 20 min, and the absorbance was measured at 517 nm using a spectrophotometer.

#### 2.7.2. ABTS Free Radical Scavenging Rate

This process was slightly revised with reference to Aloulou et al. [4]. A mixture of 7 mmol/L ABTS solution and 2.45 mmol/L K_2_S_2_O_8_ at a concentration of 7 mmol/L ABTS solution and 2.45 mmol/L K_2_S_2_O_8_ was allowed to stand for 12 h at 25 °C protected from light, and the ABTS working solution was then prepared by diluting the solution with phosphate buffer; an absorption value of 0.7 ± 0.02 was obtained at 734 nm. A volume of 0.5 mL of the sample was taken to be tested and mixed with 2 mL of ABTS storage solution, then reacted for 5 min at 25 °C away from light; the absorbance at 734 nm was measured using a UV spectrophotometer.

#### 2.7.3. Total Reducing Power (TRP) Determination

The reducing power was determined by the potassium ferricyanide method [19]. The specific method is as follows: 2 mL of 0.2 mol/L phosphate buffer and 2 mL of potassium ferricyanide solution (1 g/100 mL) were added to 0.2 mL of sample solution. The mixture was then incubated in a water bath at 50 °C for 20 min. Thereafter, the reaction was stopped with 2.5 mL of TCA (10 g/100 mL). Following centrifugation at 6000× *g* r/min for 10 min, the resulting 2.5 mL of upper layer was mixed with 2.5 mL of deionized water and 0.5 mL of ferric chloride (100 mg/100 mL). The mixture’s absorbances were measured at 700 nm.

### 2.8. Electronic Nose Analysis

The assay was performed using the PEN3.5 electronic nose (AIRSENSE Analytics, Schwerin, Germany), following the method of Zhu et al. [20], with minor modifications. A 15 mL sample was placed into a 50 mL centrifuge tube. The tube was then closed and the headspace gas was allowed to equilibrate for 30 min. During the measurement phase, the headspace gas passes through the sensor array at a rate of 300 mL/min, which induces a change in the conductivity of the sensor. The response of the sensor is defined as the ratio of conductivity G/G_0_ (G0 and G are the conductivity of the sensor before and after contact with the gas sample, respectively). The duration of the measurement phase is 120 s, which is deemed sufficient time for the sensor to reach a stable response value. At the conclusion of the measurement, the acquired data is stored for future reference. Subsequent to this, a standby phase commences, with a duration of 100 s. During this phase, the presence of clear air serves to extract residual volatiles from both the sensor and the circuitry. Thereafter, the signal reverts to the baseline value prior to the initiation of the subsequent test. It is imperative to maintain an ambient temperature of 25 °C to minimize experimental error.

### 2.9. Electronic Tongue Analysis

The assay was performed using the ASTREE electronic tongue (Alpha MOS, Toulouse, France), following the method of Zhu et al. [20]. A 30 mL sample was injected into a 50 mL sample cup for testing, and the instrument was first subjected to three cleaning runs, each lasting 90 s, 120 s, and 120 s. Subsequent to the cleaning process, the sensor balance was rested for 30 s prior to measurement, and the sample was then measured for 30 s. The reference solution was then returned to, in order to measure the retentate value. Subsequently, the sensor is subjected to a dual cleaning cycle, following which the reference solution is reintroduced to recalibrate the flavor back value, with this procedure being repeated for each measurement. It is important to note that the sensor cleaning process is automated and occurs after each measurement. The potential difference between each individually coated sensor and the Ag/AgCl reference electrode in equilibrium was measured and recorded at room temperature. Four replicate measurements were performed for each sample. Due to the occurrence of instability, the initial measurement cycle was discarded, and only the subsequent three stable sensor responses were utilized as raw data.

### 2.10. Sensory Evaluation of SBK

The sensory evaluation of SBK was conducted by a panel of ten subjects (aged 22–30 years; five females and five males) from the School of Food, Nutrition and Engineering at Northwest Agriculture and Forestry University (NWAFU). The panelists were tasked with the assessment of the kombucha samples based on their smell, color, form, taste, and overall acceptability. A 9-point hedonic scale was utilized to ascertain the participants’ preferences for the kombucha samples, with acceptability ranging from 9 (highest) to 1 (lowest). Prior to the commencement of the study, the participants were provided with a detailed briefing to ensure their informed consent and the option to withdraw from the study at any time. The kombucha samples were stored in a refrigerated environment at 4 °C and consumed at room temperature. Each sample was served in a clear 50 mL plastic cup. During the evaluation process, the palate was cleansed with purified water. Finally, after tasting all samples, participants were asked to rank all four samples from favorite to least favorite.

The experiment was conducted in accordance with the tenets of the Declaration of Helsinki of 1975, and appropriate measures were taken to address any risks that might arise. All group members participated voluntarily after being informed of the risks, were given the right to information and consent, and their privacy was adequately protected.

### 2.11. Statistics and Analyses

All experimental data were processed for three cycles, after which the processed data were expressed as mean ± standard deviation. The antioxidant activity, quality analysis, and so on were analyzed by one-way analysis of variance (ANOVA) using Tukey’s analysis (*p* < 0.05), with SPSS Statistics 27 (IBM, Armonk, NY, USA) software. The correlation between antioxidant activity and compound concentration was calculated using Pearson’s coefficient with Origin 2018 (OriginLab, Northampton, MA, USA), whilst principal component analysis (PCA) of e-tongue and e-nose was performed using PCA plug-in in Origin, employing correlation matrix coefficients. The images were plotted using Origin 2024 (OriginLab, Northampton, MA, USA) and GraphPad Prism10 (GraphPad Software, Boston, MA, USA).

## 3. Results and Discussion

### 3.1. pH, Total Acid, and TSS

**Figure 2:**
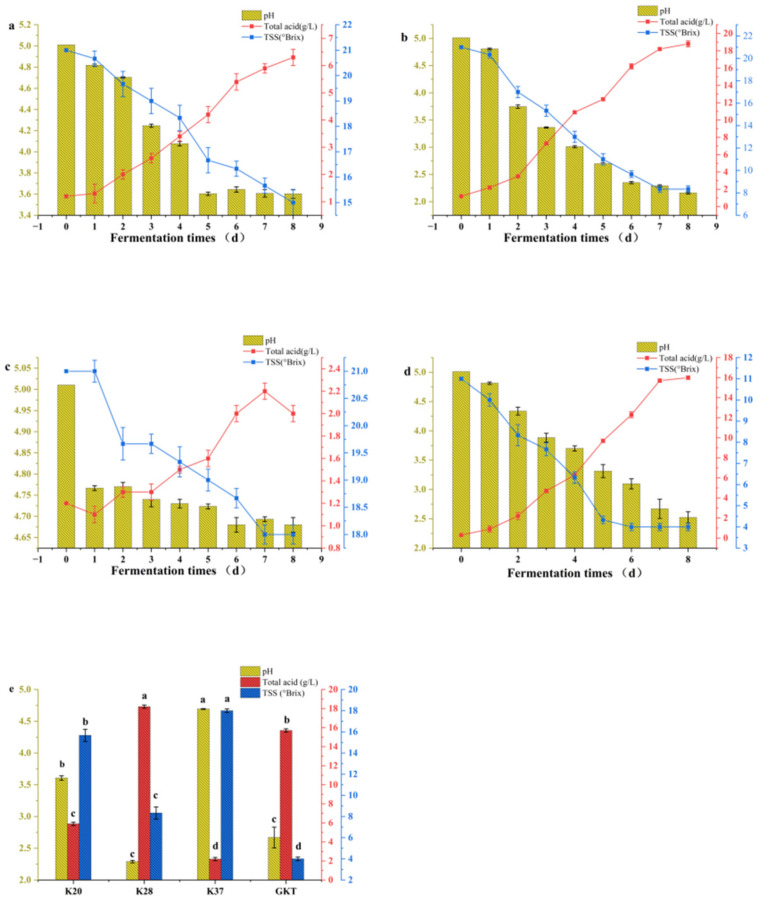
Changes of pH, total acid, and TSS during fermentation of kombucha. (**a**) Changes of K20, (**b**) changes of K28, (**c**) changes of K37, (**d**) changes of GKT. (**e**) On day 7, each sample was analyzed for multiple comparisons of pH, total acid, and TSS. The utilization of different letters in the Figure 2(e) is indicative of significant differences (*p* < 0.05).

The results of pH, total acid and TSS for each sample during fermentation are shown in Figure 2. During the fermentation process, pH has been shown to exert a significant influence on the sensory characteristics of the final product, and it is known that pH changes are associated with the activity of microorganisms in kombucha; thus, pH can be considered an important indicator of kombucha fermentation [21]. In this research study, the decline of the pH value during the kombucha fermentation process was observed. Initial pH levels were measured at 5.01 for samples of sea buckthorn kombucha on day 0, and these decreased to 3.60, 2.16, and 4.68 at the conclusion of the fermentation period. GKT samples demonstrated a pH decrease to 2.52. Consistent with the phenomenon under observation, several other studies have been conducted, which provide support for our observations. In one such study [22], kombucha tea was fermented and observed to undergo a significant pH decline from 5.0 to 2.5 over the fermentation period (6–10 days). In a study by Sharifudin et al. (2021) [23], it was observed that fermentation of kombucha using papaya as a substrate showed no significant change in pH as compared to the traditional method. In addition, our experimental results support this observation, with K28 showing no significant change in pH compared to GKT at equivalent temperatures (*p* > 0.05) (refer to Figure 2e). It is also worth noting that the pH of sea buckthorn kombucha varied at different temperatures (*p* < 0.05), which suggests that fermentation at different temperatures can affect the pH of kombucha.

In kombucha fermentation, saccharomyces converts sugar to ethanol and carbon dioxide, and acetobacter oxidizes ethanol to acetic acid, which is the main organic acid produced in kombucha fermentation [24]. It was observed that the concentration of total acid increased in all the samples during the 7 days of fermentation, with the highest increase in K28 at 17.6 g/L, followed by GKT at 15.7 g/L, and the lowest in K37 at only 0.8 g/L. This finding suggests that the total acid content of kombucha is influenced by variations in fermentation temperature. The growth of kombucha as SCOBY is acutely sensitive to temperature. In conditions that are suboptimal, the yeast is unable to convert sucrose into ethanol and other substances in a timely manner, resulting in inadequate conditions for the survival of acetic acid bacteria. This ultimately leads to the death of acetic acid bacteria. due to a lack of nutrients, thus terminating the fermentation reaction. Consequently, it can be concluded that the maintenance of an optimal temperature is of paramount importance for the successful cultivation of kombucha [21].

TSS is typically employed to denote the aggregate composition of all dissolved compounds within a liquid matrix. In the context of kombucha, the predominant TSS components are typically sugars, as illustrated in Figure 2. After 7 days of fermentation, it was observed that the TSS content in the GKT was reduced by 63.6%, 61.9% for K28, and a minimum of 14.3% for K37. In the context of kombucha, the decline in soluble solids can be attributed to the decomposition of sucrose by yeast. Specifically, yeast enzymes catalyze sucrose decomposition into fructose and glucose, with the concomitant generation of ethanol via the glycolytic pathway. Subsequent to this, Bacillus acetobacter oxidizes ethanol to produce acetic acid via ethanol dehydrogenase and aldehyde dehydrogenase, resulting in the production of water and carbon dioxide [24]. Kitwetcharoen et al. [25] also observed a reduction in sucrose levels in kombucha as fermentation time increased. Sharifudin et al. [23] conducted an experiment using papaya as a substrate for the fermentation of kombucha, finding that the soluble solids of papaya pulp kombucha decreased by 32.6%, while that of papaya leaf kombucha decreased by 31.03%.

### 3.2. CIELab Colors

**Figure 3:**
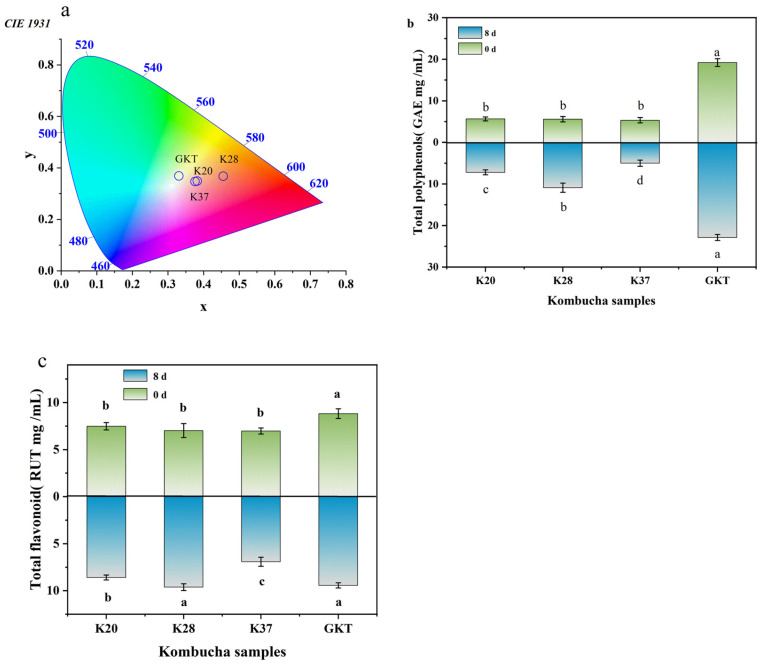
Bioactive components and color maps of kombucha fermentation for 0 d and 8 d, (**a**) color maps, (**b**) total phenolic content, and (**c**) total flavonoid content of kombucha samples. Different lowercase letters indicate significant differences between samples during fermentation. GAE: gallic acid equivalent, RUT: rutin equivalent.

Color, as a visual quality characteristic, is an influential factor in consumer choice of food products, which may lead to some changes in color due to different fermentation products. The results are shown in Figure 3a. SBK and GKT had different color values. Following eight days of fermentation, K37 and K20 exhibited the lowest brightness values L*, 65.54 ± 0.01 and 65.70 ± 0.18, with positive red a* values, 15.43 ± 0.22 and 16.89 ± 0.12, with no significant difference between the yellow b* values (*p* > 0.05). A comparison between GKT and K28 revealed that the former had a brightness of 67.18 ± 0.04, while the latter was 67.20 ± 0.08, with no significant difference between the two (*p* > 0.05). However, the a* values were found to be −6.93 ± 0.49 and 34.14 ± 1.07 in the former and the latter, respectively, indicating opposite color trends. A statistically significant difference was observed between these two values (*p* < 0.05), which is consistent with the findings reported in the study of Zijuan fermented kombucha tea [14], and there was a significant difference between the values of yellow b* (*p* < 0.05), with values of 13.14 ± 0.41 and 35.73 ± 1.40. The color difference value ∆E*ab can also better respond to the fact that there is a big difference between the two colors, with values of 10.36 ± 0.63, 33.02 ± 1.54, and 8.68 ± 0.66 for K20, K28, and K37, respectively. At this time, the overall appearance of K28 was clear but not translucent, with a slightly reddish golden yellow color. A biofilm was suspended on the surface and there was a large amount of precipitate at the bottom, which was detected as sea buckthorn precipitate.

### 3.3. Bioactive Ingredient

#### 3.3.1. Total Polyphenols

The TP of SBK and GKT final fermentation is shown in Figure 3b. The study showed that the TP content increased in all the fermented kombucha samples. The phenolic content of GKT at the end of fermentation was higher than that of the SBK group, probably caused by the high number of natural polyphenols in the tea [12]. Despite the initial similarity in TP content amongst the samples, a notable divergence in TP concentration emerged as the fermentation process reached its conclusion. Specifically, the TP levels in the K28 sample exhibited a marked increase over those observed in the K20 and K37 samples (*p* < 0.05), The mean values for TP concentration in the K28, K20 and K37 sample were 10.92 ± 1.10 (GAE mg/mL), 7.22 ± 0.60 (GAE mg/mL), and 5.01 ± 0.75 (GAE mg/mL). Appropriate temperature during fermentation promotes enzyme activity, and thus biotransformation of phenolic compounds, increasing total phenolics and forming smaller molecular weight phenolic compounds [5]. These results indicate that fermentation at 28 °C is more favorable and produces more phenolics compared to the other two temperatures.

#### 3.3.2. Total Flavonoids

As demonstrated in Figure 3c, the TF content of the final fermentation of SBK and GKT is shown. At the initial stage of fermentation, TF in GKT was slightly higher than that in the SBK group, which may be related to the dilution carried out during the preparation of sea buckthorn juice. A rise in TF content was evident in all samples at the conclusion of fermentation, with K28 exhibiting a higher content than K20 and K37, ultimately measured at 9.62 ± 0.36 RUT mg/mL. No statistically significant difference was found between the TF contents of K28 and GKT (*p* > 0.05). This finding indicates that the incorporation of sea buckthorn does not impact the TF content of kombucha maintained at a constant fermentation temperature. Tu et al. [26] demonstrated that the increase in TF content is associated with the organic acids produced by fermentation, and lower pH release binds to more flavonoids. Furthermore, the secretion of enzymes such as β-glucosidase by microorganisms during the fermentation process has been demonstrated to be a significant factor in the increased secretion of flavonoid compounds. It can thus be concluded that the TF content of kombucha is indirectly affected by fermentation temperature, due to the effect this has on the total acid content of the substance.

#### 3.3.3. Determination of Antioxidant Activity

**Figure 4:**
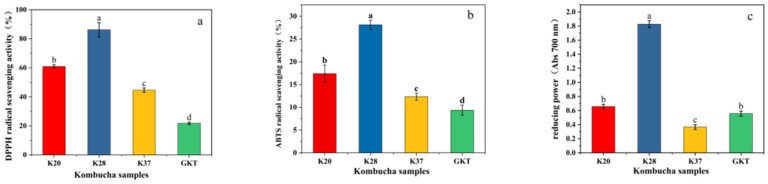
Free radical scavenging activity of kombucha fermented for 8 days: (**a**) DPPH free radical scavenging efficiency (%), (**b**) ABTS free radical scavenging efficiency (%), and (**c**) reducing power (measured at Abs 700 nm). Different lowercase letters denote statistical difference (*p* < 0.05).

The antioxidant capacity of four kombucha samples was analyzed using three different antioxidant evaluation methods, specifically DPPH, ABTS, and TRP, and the results are displayed in Figure 4. It is evident from the data that the antioxidant activity of SBK was significantly higher than that of GKT at the end of fermentation (*p* < 0.05), with K28 demonstrating the highest antioxidant activity. The scavenging rates of DPPH and ABTS free radicals in K28 at 8 d were 86.23% and 28.13%, respectively, which were the highest among all kombucha samples, and more than four and three times the GKT values, respectively. The TRP exhibited a positive correlation with the antioxidant capacity, i.e., the higher the reducing power value, the higher the antioxidant capacity [27]. As demonstrated in Figure 4c, the TRP of K28 exhibited the highest value among all kombucha samples at 1.83 ± 0.08, which was more than three times the values of K20 and GKT, and more than five times the value of K37. The higher antioxidant activity in the SBK samples suggests a possible synergy with the high Vc content in sea buckthorn and possible synergism with the organic acid compounds, especially acetic acid, produced after fermentation. Another study showed that the fermentation process of kombucha tea produces polyphenolic compounds, gluconic acid, and glucuronic acid with strong antioxidant activity [28].

### 3.4. Analysis of Electronic Nose and Tongue Results

**Figure 5:**
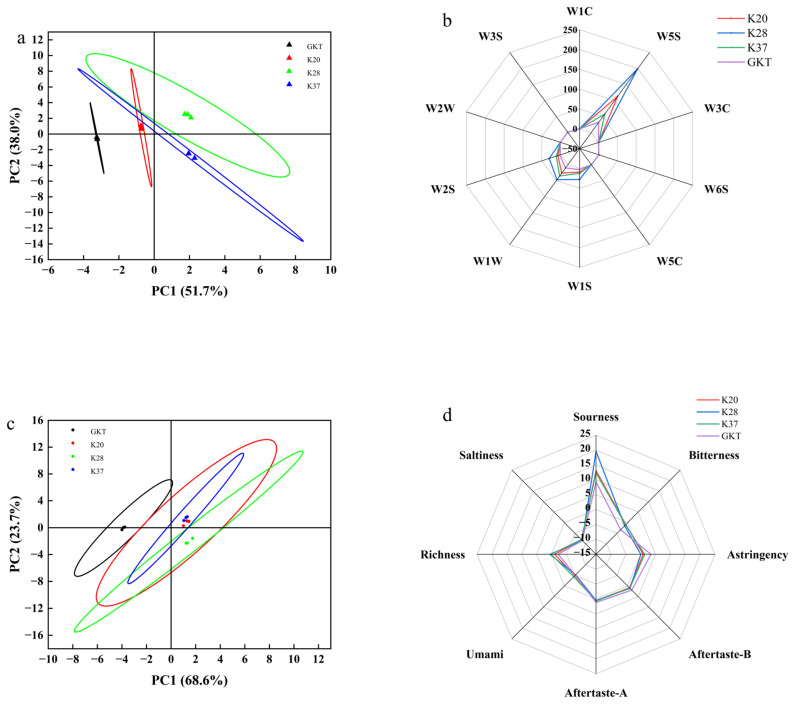
Results of e-nose and e-tongue of different kombucha samples at the end of fermentation. (**a**) E-nose PCA analysis, (**b**) electronic nose radar chart, (**c**) e-tongue PCA analysis, (**d**) electronic tongue flavor analysis radar chart.

Electronic noses have been developed for the purpose of detecting volatile compounds in food products, with the additional capability of providing real-time information about different classes of compounds (Jiang et al., 2015) [29]. These data can then be visualized using PCA (principal component analysis). The findings demonstrated that the initial two principal components accounted for 89.7% of the total variance among the four kombucha samples, with PC1 and PC2 contributing 51.7% and 38.0% of the variance, respectively (Figure 5a). As demonstrated in the figure, the sample GKT was located in the lower left region and the sample SBK was located in the right region, with no intersection between the two, indicating a significant difference between GKT and SBK (*p* < 0.05); the electronic nose demonstrated a high capacity for distinguishing between the two samples. However, the different SBKs were cross-linked with each other and could not be well distinguished. As demonstrated in Figure 5b, the degree of contribution of various components of odor to the odor of kombucha can be visualized. The sensor signals in SBK were stronger than those in GKT, with K28 having the strongest odor, and the response values of W5S (mainly sensitive to nitrogen oxides), W1S (sensitive to methyl compounds), W1W (sensitive to inorganic sulfides), and W2S (sensitive to alcohols, aldehydes and ketones) were significantly higher compared with those of GKT. This finding suggests that the flavoring substances of SBK are primarily nitrogen oxides, methyls, inorganic sulfides, alcohols, and aldehydes and ketones.

**Figure 6:**
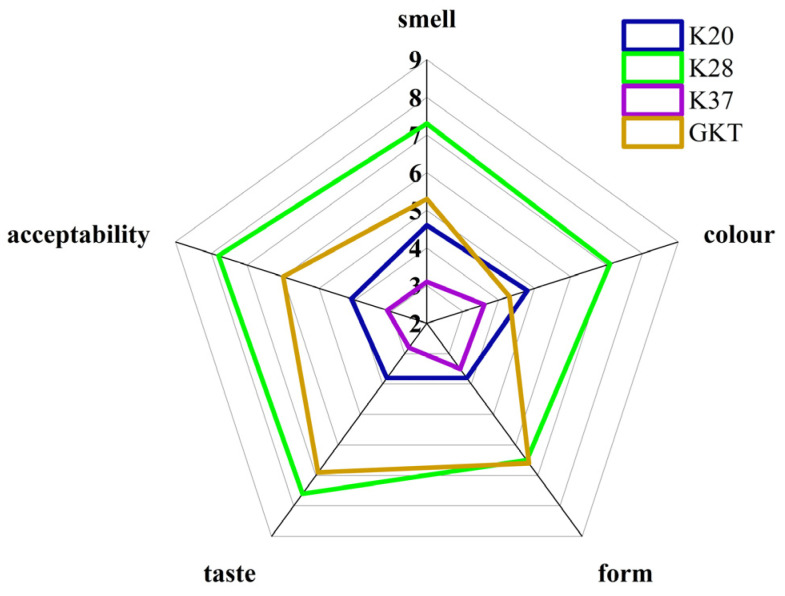
Sensory property evaluation on kombucha beverages.

The electronic tongue not only mimics the human tongue, but also can present flavors more sensitively, automatically and impartially than human tasters. The flavor profile of each sample was analyzed by electronic tongue to determine the contribution of each flavor. As demonstrated in Figure 5c, the variance contribution of PC1 was 68.6%, PC2 was 23.7%, and the cumulative variance contribution was 92.3%. This finding indicates an absence of correlation between the SBK and GKT tea samples, which is consistent with the e-nose results, suggesting that the volatile profiles of the SBK and GKT samples are significantly divergent. It is evident that the PCA captured over 85% of the variability in the kombucha samples, thereby highlighting the ability of the method to encapsulate the predominant characteristics of the volatile components present in diverse kombucha. As shown in Figure 5d, SBK has a higher intensity of sourness in terms of more prominent sourness, related to higher acetic acid produced by fermentation [30], which is consistent with the phenomenon of sensory evaluation (Figure 6). The second was higher than GKT in richness and bitterness indicators, caused by the different metabolites produced by the different fermentation substrates [31]. On the contrary, GKT was higher in astringency than SBK, mainly due to polyphenols and catechins produced by the tea and fermentation. Overall, the e-tongue and e-nose demonstrated a high level of accuracy in distinguishing between SBK and GBK, but were unable to differentiate between kombucha beverages at varying fermentation temperatures.

### 3.5. Sensory Evaluation

Sensory characteristics such as odor, color, morphology, taste, and acceptability of the four kombucha samples were evaluated and the results are shown in Figure 6.

For SBK, K28 had no statistically significant difference (*p* > 0.05) with GKT in appearance and morphology, but it had a significant difference (*p* < 0.05) with K20 and K37, which could be caused by too low or too high temperatures unfavorable to kombucha fermentation. Compared with the color of GKT, the color of SBK was darker; in particular, the color of K28 was more translucent towards orange-red, with the highest score of 7.1 among the four samples, which was the most preferred sample in terms of color in the sensory preference test. In addition, in terms of odor and taste, GKT was higher than the other two groups, but slightly lower than K28, which had a moderate acidity and a strong aftertaste with a more three-dimensional taste, while the GKT group was relatively more acidic and astringent, which may be related to the tea polyphenols and catechins in the tea leaves. In the acceptability test, 90% of these testers showed unacceptability for K37, with a minimum score of only 3.1, while GKT and K28 were more acceptable overall, but the latter was higher. Overall, among the four kombucha beverages in this study, SBK fermented at 28 °C scored the highest on all indicators.

### 3.6. Correlation Analysis Between Antioxidant Parameters and Bioactivities

**Figure 7:**
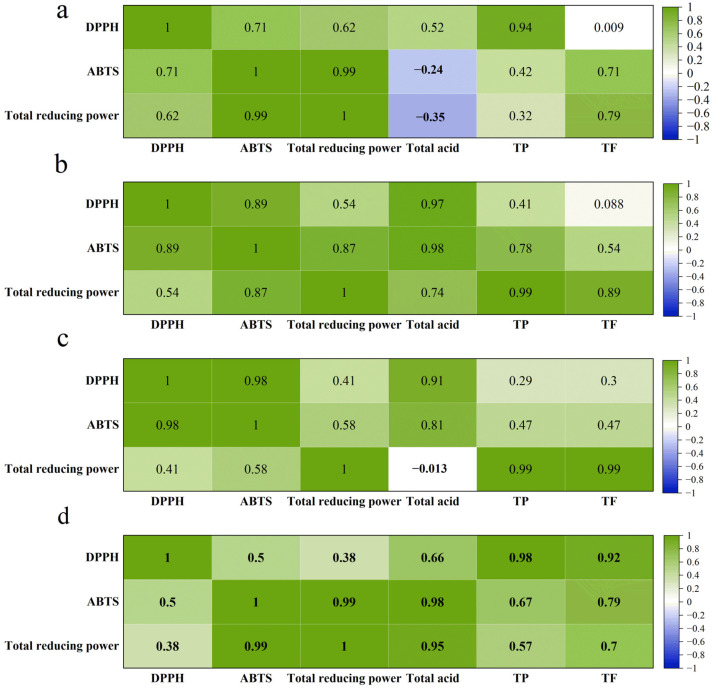
Heat map analysis of correlation between different antioxidant parameters and biological activities using Pearson’s coefficient. (**a**) K20, (**b**) K28, (**c**) K37, and (**d**) GKT.

The results of the correlation between the antioxidant properties (DPPH, ABTS, and TRP) and the biological activities (total acids, TP, and TF) of the four kombucha under different fermentation conditions are shown in Figure 7.

Correlation between DPPH radicals and bioactive compounds in SBK, the results are shown in Figure 7. A high correlation (r = 0.94) was found between DPPH and TP content in K20; moreover, a correlation was also found between DPPH and the total acids of the other two types of kombucha (K28 and K37) (r = 0.97 and r = 0.91). These results suggest that total acids and phenolic substances contribute to the elimination of DPPH free radical values. Many studies have shown that sea buckthorn contains a high amount of phenolics that play an important role in enhancing antioxidant activity, which is consistent with the results of the present study [32]. Interestingly, we also observed a significant correlation between total acids and TP and DPPH in GKT (r = 0.66 and r = 0.98, *p* < 0.05), which is in line with the findings of Malbasa et al. [33] regarding the addition of tea dregs to kombucha fermentation, suggesting that addition of a certain amount of tea during fermentation is more favorable for antioxidant activity.

For the correlation between ABTS and bioactive compounds in kombucha, there was a correlation between ABTS values and TP (r = 0.42, r = 0.78, and r = 0.47) and TF (r = 0.71, r = 0.54, and r = 0.47) in SBK fermented at different temperatures, and the results indicated that flavonoids in sea buckthorn also play a role in antioxidant. In addition, TP (r = 0.67) and TF (r = 0.79) were significantly correlated (*p* < 0.05) with ABTS values in GKT fermented.

For the correlation between TRP values and bioactive compounds in kombucha, it was found that there was some correlation between TRP and TF content in K20 (r = 0.79) and significant correlation between TP (r = 0.99 r = 0.89) and TF (r = 0.99 r = 0.99) in K28 and K37 SBK *p* < 0.05, while there was some correlation between TRP and GKT values (r = 0.57 and r = 0.70).

As demonstrated in Figure 3b, a significant disparity in the polyphenol content of GKT and SBK was observed. This discrepancy can be attributed, primarily, to the elevated polyphenol content inherent in green tea. However, a contradictory finding emerged from the antioxidant activity experiments and correlation analyses, despite the prevailing consensus that tea polyphenols and flavonoids are associated with antioxidants. However, certain components of the raw material, like Vc, have been identified as a significant component in some fruits, with sea buckthorn being a notable example. It is plausible that the higher antioxidant levels observed in SBK may be attributable to its high Vc content. Green tea and sea buckthorn have been identified as potential sources of phenolic and Vc compounds, which may play a significant role in the strong antioxidant capacity of kombucha tea products. However, the Vc content of the fermentation broth was not determined in this article and was not explored in more depth. Consequently, a subsequent phase of research will entail investigating whether the fermentation of kombucha tea can preserve the Vc in sea buckthorn, representing a novel research direction that merits further exploration.

In conclusion, more phenolic and flavonoid compounds in SBK contributed to the DPPH, ABTS, and TRP values of kombucha compared to GKT. Among them, phenolic compounds may be the main compounds responsible for the high antioxidant activity of SBK.

## 4. Conclusions

In this study, a novel kombucha beverage was prepared for the first time using sea buckthorn juice. The differences in sensory assessment, substance composition, and antioxidant properties of kombucha at different temperatures were analyzed. The results demonstrated that SBK produced at varying temperatures exhibited significant alterations in fundamental physicochemical indices and antioxidant activity. Notably, fermentation at 28 °C led to a substantial increase in total acid, TP, and TF values in SBK beverages, which were characterized by high antioxidant properties. The increased acidity of SBK resulted in lower pH values, but within acceptable limits. Electronic nose analysis revealed that the predominant flavor compounds contributing to SBK were nitrogen oxides, methyl groups, inorganic sulfides, alcohols, aldehydes, and ketones. Furthermore, the e-tongue results indicated that SBK had a more pronounced acidic flavor, while GKT had a higher level of astringency. Despite higher levels of TP and TF being observed in GKT compared to SBK, the antioxidant test yielded divergent results, a phenomenon that may be associated with the elevated Vc content characteristic of sea buckthorn. Consequently, sea buckthorn juice with added sea buckthorn is hypothesized to have higher bioactivity and antioxidant properties compared to traditional kombucha tea. Furthermore, the optimal temperature for the fermentation of kombucha tea is hypothesized to be 28 °C, for the preparation of a kombucha beverage. However, further exploration is required to elucidate the other active functions of sea buckthorn kombucha.

## Data Availability

The original contributions presented in the study are included in the article; further inquiries can be directed to the corresponding author.

## Abbreviations

Acronyms covered in this article are preceded by full names and abbreviated in parentheses. Green tea kombucha (GKT); sea buckthorn kombucha fermented at 20 °C (k20); sea buckthorn kombucha fermented at 28 °C (k28); sea buckthorn kombucha fermented at 37 °C (k37); vitamin C (Vc); sea buckthorn kombucha (SBK); Symbiotic Culture of Bacteria and Yeast (SCOBY); total reducing power (TRP); total phenolic (TP); total flavonoid (TF); gallic acid equivalent (GAE); rutin equivalent (RUT); principal component analysis (PCA); optical density (OD); total soluble solids (TSS).
